# Tumor regression grade predicts recurrence-free survival in intermediate-advanced hepatocellular carcinoma after conversion therapy

**DOI:** 10.3389/fimmu.2025.1704239

**Published:** 2025-10-30

**Authors:** Haili Zhang, Tongshu Yang, Luping Huang, Bo Li, Hongwei Xu, Yonggang Wei

**Affiliations:** ^1^ Division of Liver Surgery, Department of General Surgery, West China Hospital, Sichuan University, Chengdu, China; ^2^ Key Laboratory of Hepatobiliary and Pancreatic Surgery, Institute of Hepatobiliary Surgery, Southwest Hospital, Third Military Medical University (Army Medical University), Chongqing, China; ^3^ Department of Nuclear Medicine, Sichuan Clinical Research Center for Cancer, Sichuan Cancer Hospital & Institute, Sichuan Cancer Center, Affiliated Cancer Hospital of University of Electronic Science and Technology of China, Chengdu, China; ^4^ State Key Laboratory of Biotherapy, Sichuan University, Chengdu, China

**Keywords:** hepatocellular carcinoma, conversion therapy, tumor regression grade, recurrence-free survival, prediction

## Abstract

**Background:**

Conversion therapy provides patients with intermediate-advanced hepatocellular carcinoma (HCC) an opportunity for radical resection, but the prognostic significance of residual tumor in resected specimens remains unclear. This study aimed to evaluate the impact of pathological tumor regression grade (TRG) on postoperative recurrence in HCC patients who underwent conversion therapy.

**Methods:**

Data from HCC patients who received conversion therapy between September 2018 and September 2022 were retrospectively collected. Patients were categorized based on TRG status: TRG1a, TRG1b, TRG2, and TRG3. Multivariate regression analyses were performed to identify independent predictors of recurrence and factors associated with optimal TRG status after resection.

**Results:**

A total of 117 patients who underwent conversion resection were enrolled. Multivariate regression analysis revealed that achieving TRG1b was not significantly associated with recurrence, whereas TRG2 (HR = 4.25, *P* = 0.012) and TRG3 (HR = 6.20, *P* < 0.001) were independently associated with an increased risk of postoperative recurrence compared to TRG1a. The combined TRG1(a&b) group demonstrated significantly longer recurrence-free survival (RFS) compared to the TRG2 (*P* = 0.0075) and TRG3 (*P* < 0.001) groups, respectively. Independent predictors for achieving TRG1(a&b) included male gender (OR = 3.44, *P* < 0.001), treatment with TACE combined with targeted therapy and immunotherapy (*P* < 0.001), pre-treatment tumor diameter of 5–10 cm (OR = 2.31, *P* = 0.012), and rapid normalization of AFP levels (OR = 0.97, *P* < 0.001).

**Conclusions:**

Achieving TRG1 status (0-10% residual tumor) following conversion therapy is associated with significantly improved RFS in patients with intermediate-advanced HCC. Male patients, tumors measuring 5–10 cm, combined TACE with systemic therapies, and rapid normalization of AFP levels are significant predictors for achieving TRG1 status.

**Clinical Trial Registration:**

ChiCTR2400088877.

## Introduction

Hepatocellular carcinoma (HCC) represents the most common primary liver malignancy and ranks as the third leading cause of cancer-related mortality worldwide (1, 2). Despite advancements in early detection and surgical techniques, the long-term survival of HCC patients remains unsatisfactory, with approximately 70% experiencing disease recurrence within five years (3, 4). Reasons behind this could be that most patients are initially diagnosed at intermediate or advanced stages, missing the window for curative liver resection, while alternative treatment options are limited (5, 6). Consequently, there is an urgent need to develop novel therapeutic strategies to improve long-term outcomes for this patient population.

In recent years, the advent of targeted therapy and immunotherapy has markedly transformed the treatment landscape for HCC (7, 8). Since 2007, targeted agents such as sorafenib and lenvatinib have been widely used in patients with advanced HCC (9, 10). Immunotherapy, which harnesses the patient’s immune system to combat tumor cells, has also emerged as a promising modality with potent anti-tumor efficacy (11, 12). Furthermore, the combination of targeted therapy and immunotherapy has established a new treatment paradigm for HCC, particularly for patients with advanced disease deemed unresectable at initial diagnosis (13). Encouragingly, a subset of these patients may qualify for radical liver resection following successful systemic treatment, an approach known as conversion therapy for HCC, which signifies a major breakthrough in managing intermediate-to-advanced HCC (14). Upon receiving radical surgery after successful conversion therapies, the pathological examination of resected specimens often reveals varying degrees of tumor necrosis, serving as a key indicator of treatment efficacy. Notably, a small proportion of patients achieve pathologic complete response (pCR), characterized by complete tumor necrosis, which has been correlated with significantly improved long-term survival (15).

However, not all patients who undergo successful conversion therapy achieve pCR. In clinical practice, major pathologic response (MPR), indicating partial tumor necrosis, is more commonly observed. The extent to which partial necrosis influences survival outcomes remains unclear, and no consensus exists on the threshold of MPR that significantly impacts post-resection recurrence (16). Regarding this issue, the Becker tumor regression grade (TRG) system, which categorizing the degree of tumor response to therapy, provides a standardized method to quantify tumor necrosis (17). while it was originally developed for gastric cancer, it has been widely adopted and validated in the assessment of treatment response in various other solid tumors following neoadjuvant therapy (e.g., lung, colorectal cancers). Its simplicity, reproducibility, and the clinical relevance of its categories (particularly the distinction of ≤10% residual tumor) make it a practical and valuable tool for initial exploratory studies in HCC, where a universally accepted, standardized TRG system in the context of HCC therapy is currently lacking. Therefore, this study aimed to investigate the prognostic significance of TRG in HCC patients undergoing conversion therapy and to determine the optimal TRG threshold associated with long-term survival benefit.

## Methods

### Study design

Data from HCC patients who visited West China Hospital of Sichuan University and received conversion treatments between September 2018 and September 2022 were retrospectively collected. The inclusion criteria were as follows: 1) diagnosis of intermediate or advanced HCC (BCLC-B or BCLC-C stage) before conversion treatments; 2) patients aged 18–85 years old; 3) patients with preserved liver function (Child-Pugh class A) and good performance status (ECOG score 0 - 1) at the initiation of treatment; and 4) subsequent surgical resection following conversion therapy with available pathological evaluation of HCC. The exclusion criteria included: 1) pathological diagnosis of mixed-type HCC (e.g., combined hepatocellular-cholangiocarcinoma); 2) patients received liver transplantation or radiofrequency ablation instead of liver resection following the systemic treatments; 3) incomplete clinical or follow-up data; and 4) recurrent HCC at the initial visit. This study was approved by the Institutional Review Board of West China Hospital of Sichuan University and conducted in comply with the Declaration of Helsinki. This study was registered in Chinese Clinical Trial Registry (ChiCTR2400088877).

### Conversion therapy and surgical resection

The conversion regimens included transcatheter arterial chemoembolization (TACE) alone, TACE combined with tyrosine kinase inhibitors (TACE+TKI), TACE combined with TKI and immune checkpoint inhibitors (TACE+TKI+ICI), and TACE combined with Bevacizumab and ICI (TACE+Bev+ICI). The TKIs involved in this study were Sorafenib, Donafenib, and Lenvatinib, while the ICIs included Camrelizumab, Sintilimab, Pembrolizumab, Durvalumab, Atezolizumab, and Tislelizumab. The finalization of patients treatment plans were determined through multidisciplinary team (MDT) discussions involving hepatobiliary surgeons, oncologists, pathologists, and radiologists. The selection of specific regimens was influenced by drug availability and patients’ financial considerations, with all decisions made following detailed physician-patient consultations.

In terms of the subsequent liver resections, the intermittent Pringle maneuver combined with low central venous pressure anesthesia was routinely employed to minimize intraoperative blood loss. Intraoperative ultrasound was utilized to delineate tumor boundaries and guide resection margins. A drainage tube was routinely placed on the transection surface after hepatectomy.

During postoperative follow-up, patients were evaluated every three months via outpatient visits. Assessments included routine blood tests, liver and kidney function tests, tumor marker assays, and imaging studies (CT, MRI, or ultrasound). Postoperative adjuvant therapy followed the pre-conversion regimen until disease recurrence was detected.

### Groups and definitions

Based on pathological evaluation of resected specimens, patients were stratified according to the Becker TRG system as follows (17): TRG1a (no residual tumor/tumor bed), TRG1b (≤10% residual tumor/tumor bed), TRG2 (10 - 50% residual tumor/tumor bed), and TRG3 (> 50% residual tumor/tumor bed). This classification system was selected for its clear, quantitative criteria and proven utility in grading neoadjuvant therapy response in gastrointestinal cancers. For HCC resection specimens with documented conversion therapy, the tumor bed (original tumor site prior to treatment) was sectioned along its longest diameter, and three-dimensional measurements were recorded. A detailed macroscopic description of the extent and proportion of necrosis and residual tumor was provided according to the 2024 edition of the China Liver Cancer Guidelines (18). Small HCCs (≤3 cm in diameter) were entirely submitted for histological processing. For tumors > 3 cm, the specimen were serially sliced at intervals of 0.5-1.0 cm along the maximal cross-section. The most representative slice demonstrating residual tumor were entirely embedded, and paired samples from the tumor bed and adjacent non-tumorous liver tissue were obtained for comparative analysis. Microscopic evaluation involved quantitative assessment of three key components within the tumor bed: viable tumor, necrotic areas, and tumor stroma (including fibrous tissue and inflammatory cells). The combined area of these three elements should total 100%. The pathology report specified the number of tissue blocks examined and the overall percentage of residual tumor were calculated as the average of the percentages of viable tumor across all sampled sections and explicitly stated in the final report.

Treatment response to conversion therapy was assessed using the Modified Response Evaluation Criteria in Solid Tumors (mRECIST) criteria (19). The extent of portal vein tumor thrombus was classified according to the Vp classification system (20). Postoperative complications were evaluated with criteria as follows: liver failure was defined per the “50–50 criteria” on postoperative day 5 (21), postoperative hemorrhage was identified by a hemoglobin drop > 3 g/dL compared to the postoperative baseline and/or any transfusion of packed red blood cells for declining hemoglobin levels (22), and bile leakage was defined according to ISGLS criteria (23). The primary endpoint of this study was to investigate the association between TRG status and postoperative prognosis. The secondary endpoint was to explore factors influencing the achievement of favorable TRG status.

### Statistical analysis

Continuous variables were summarized using median and range, while categorical variables were presented as number and percentages. Group comparisons were performed using the Kruskal-Wallis H test or Chi-square test, as appropriate. Survival analysis was conducted using the Kaplan-Meier method, and differences between groups were compared with the log-rank test. Multivariate analyses were performed using Cox proportional hazards models to identify independent prognostic factors for recurrence-free survival (RFS), with results reported as hazard ratios (HR) with 95% confidence intervals (CI). Factors associated with TRG status were identified using multivariate logistic regression analysis and were presented as odds ratios (OR) with 95% CI. A two-sided *P* value < 0.05 was considered statistically significant. All analyses were performed using SPSS software (version 24.0, IBM Corp.) and R software (version 4.2.2).

## Results

### Patient characteristics

**Figure 1:**
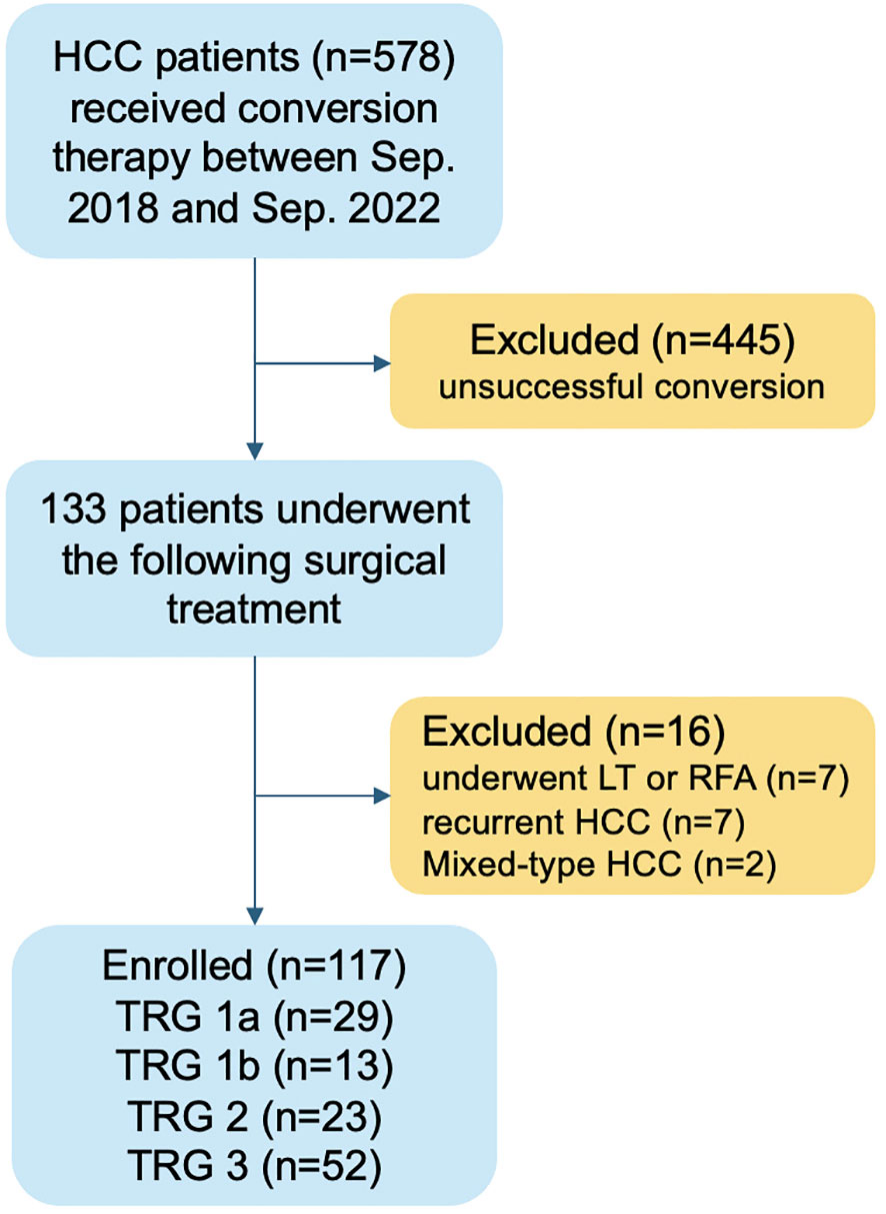
Flow chart of patients selection. HCC, hepatocellular carcinoma; LT, liver transplantation; RFA, radiofrequency ablation; TRG, tumor regression grade.

A total of 578 patients with intermediate-advanced HCC received conversion therapy during the study period. Among them, 133 (23.0%) subsequently underwent surgical treatment. After screening for exclusion criteria, 117 patients who received liver resection were included in the final analysis (**Figure 1**).

The baseline characteristics of the enrolled patients are summarized in **Table 1**. he median age was 53 years, and the majority were male (88.9%). Active hepatitis B virus (HBV) infection was present in 42.7% of patients before conversion therapy, and 32.5% had portal hypertension. According to the BCLC staging system, 53.0% and 47.0% of patients were classified as stage B and C, respectively. The median alpha-fetoprotein (AFP) level before conversion therapy was 14,070.5 ng/mL, and the median protein induced by vitamin K absence or antagonist-II (PIVKA-II) level was 14,047.7 mAU/mL. Following conversion therapy, the median AFP level decreased significantly to 82.1 ng/mL, and the median PIVKA-II level decreased to 63.3 mAU/mL. The median tumor size also decreased from 8.7 cm to 6.7 cm after treatment. Furthermore, a reduction in tumor number was observed (59.8% vs. 27.4% with multiple lesions). For patients positive for HBV DNA, oral antiviral therapy was administered throughout conversion treatment, leading to a significant reduction in the HBV DNA positivity rate (42.7% vs. 17.9%). According to mRECIST criteria, all included patients achieved at least disease control status. The most common conversion regimen was TACE+TKI+ICI, followed by TACE alone, with TACE+Bev+ICI being the least frequently used.

**Table 1:** Demographic characteristics of patients before and after conversion therapy.

Variable	Total (n=117)	TRG 1a (n=29)	TRG 1b (n=13)	TRG 2 (n=23)	TRG 3 (n=52)	*P* value
Age, (years)	53 (20–85)	56 (33-85)	59 (32-71)	52 (30-68)	52 (20-77)	0.215
Male	104 (88.9)	27 (93.1)	13 (100)	19 (82.6)	45 (86.5)	0.225
BMI	23.1 (17.0-36.4)	23.1 (18.4-27.1)	24.1 (19.9-29.1)	21.5 (16.9-27.3)	23.2 (17.7-36.4)	**0.035**
Medical history						
HBP	25 (21.4)	5 (17.2)	3 (23.1)	3 (13.0)	14 (26.9)	0.357
COPD	6 (5.1)	3 (10.3)	0 (0)	0 (0)	3 (5.8)	0.493
DM	16 (13.7)	5 (17.2)	4 (30.8)	2 (8.7)	5 (9.6)	0.164
Portal hypertension	38 (32.5)	14 (48.3)	3 (23.1)	5 (21.7)	16 (30.8)	0.159
Hypersplenism	26 (22.2)	9 (31.0)	4 (30.8)	4 (17.4)	9 (17.3)	0.115
BCLC stage						**0.041**
B	62 (53.0)	11 (37.9)	5 (38.5)	11 (47.8)	35 (67.3)	
C	55 (47.0)	18 (62.1)	8 (61.5)	12 (52.2)	17 (32.7)	
PVTT classification						0.618
Vp1	3 (2.6)	0 (0)	1 (16.7)	1 (9.1)	1 (7.7)	
Vp2	7 (5.9)	5 (29.4)	1 (16.7)	1 (9.1)	0 (0)	
Vp3	20 (17.1)	7 (41.2)	2 (33.3)	4 (36.4)	7 (53.8)	
Vp4	10 (8.5)	3 (17.6)	1 (16.7)	4 (36.4)	2 (15.4)	
HVTT	7 (5.9)	2 (11.8)	1 (16.7)	1 (9.1)	3 (23.1)	
Pre-AFP (ng/mL)*	473.6 (1-551261)/(20.8-1210)	1210.0 (3-551261)/(292-13120)	55.9 (2-3491)/(17.9-1210)	340.0 (7-174903)/(18.2-2476)	299.0 (1-105389)/(9.5-1210)	**0.029**
Pre-PIVKA-II (mAU/mL)	3216.0 (0.9-75000)	7318.5 (14-75000)	5498.0 (62-75000)	1219.0 (44-75000)	2853.0 (0.9-75000)	0.534
Pre-HBVDNA+	50 (42.7)	15 (53.6)	5 (38.5)	12 (52.2)	18 (35.3)	0.335
Pre-tumor size (cm)	8.7 (1-20)	9.5 (3.5-19.4)	9.3 (5.3-15.8)	8 (3.8-14.8)	7.7 (1-20)	0.816
Pre-tumor number						0.546
single	47 (40.2)	9 (31.0)	7 (53.8)	9 (39.1)	22 (42.3)	
multiple	70 (59.8)	20 (69.0)	6 (46.2)	14 (60.9)	30 (57.7)	
Therapy regimen						0.000
TACE	39 (33.3)	4 (13.8)	1 (7.7)	5 (21.7)	29 (55.8)	
TACE+TKI	21 (17.9)	5 (17.2)	1 (7.7)	6 (26.1)	9 (17.3)	
TACE+TKI+ICI	47 (40.2)	14 (48.3)	11 (84.6)	10 (43.5)	12 (23.1)	
TACE+Bev+ICI	10 (8.5)	6 (20.7)	0 (0)	2 (8.7)	2 (3.8)	
Post-AFP (ng/mL)*	4.2 (0-2286)/(2.5-14.1)	2.5 (0-112)/(1.7-4.1)	4 (1-9)/(3.1-5.0)	6.3 (1-341)/(2.9-16.8)	6.6 (1-2286)/(3.0-29.9)	**0.001**
Post-PIVKA-II (mAU/mL)	27.0 (0-645)	28.0 (11-461)	21.0 (0-61)	31.0 (9-494)	27.0 (8-645)	0.718
Post-HBVDNA+	21 (17.9)	2 (6.9)	1 (7.7)	1 (4.3)	17 (32.7)	**0.003**
Post-tumor size (cm)	6.9 (0.6-23)	5 (1.2-13.5)	8 (2.9-13.5)	5.5 (0.8-12)	7.3 (0.6-23)	0.209
Post-tumor number						**0.018**
single	85 (72.6)	25 (86.2)	10 (76.9)	11 (47.8)	39 (75.0)	
multiple	32 (27.4)	4 (13.8)	3 (23.1)	12 (52.2)	13 (25.0)	
mRECIST						0.000
CR	10 (8.5)	10 (34.5)	0 (0)	0 (0)	0 (0)	
PR	38 (32.5)	12 (41.4)	5 (38.5)	8 (34.8)	11 (21.2)	
SD	69 (59.0)	7 (24.1)	8 (61.5)	15 (65.2)	39 (75.0)	

*Report AFP as median (range)/(interquartile range).

BMI, body mass index; HBP, high blood pressure; COPD, chronic obstructive pulmonary disease; DM, diabetes mellitus; BCLC, Barcelona Clinic Liver Cancer; PVTT, portal vein tumor thrombus; HVTT, hepatic vein tumor thrombus; Pre-, pre-therapy; Post-, post-therapy, but before surgery; AFP, Alpha fetoprotein; PIVKA-II, protein induced by vitamin K absence-II; TACE, transcatheter arterial chemoembolization; TKI, tyrosine kinase inhibitors; ICI, immune checkpoint inhibitors; Bev, Bevacizumab; mRECIST, modified response evaluation criteria in solid tumors; CR, complete response; PR, partial response; SD, stable disease. The bold values are statistically significant.

### TRG as a prognostic factor for recurrence after successful conversion therapy

**Table 2** presents the univariate and multivariate analyses of factors associated with postoperative recurrence. Univariate analysis identified age [HR = 0.54; 95% CI (0.30 ~ 0.96), *P* = 0.036], TRG classification, microvascular invasion (MVI) [HR = 2.10; 95% CI (1.23 ~ 3.58), *P* = 0.007] and satellite nodules [HR = 2.08; 95% CI (1.18 ~ 3.65), *P* = 0.011] were significant factors influencing postoperative recurrence. However, in the multivariate analysis, only TRG remained an independent risk factor. Specifically, compared to TRG1a, achieving TRG1b [HR = 2.20; 95% CI (0.55 ~ 8.22), *P* = 0.266] was not associated with increased recurrence risk, whereas TRG2 [HR = 4.25; 95% CI (1.37 ~ 13.17), *P* = 0.012] and TRG3 [HR = 6.20; 95% CI (2.11 ~ 18.26), *P* < 0.001] were significantly associated with higher recurrence risks. In addition, the subgroup analyses (**Supplementary Figure S1**) based on HBV status, cirrhosis severity, and systemic agents demonstrated that the prognostic effect of TRG was consistent and significant (log-rank *P* < 0.05 for TRG1 vs. TRG2 or TRG3; log-rank *P* > 0.05 for TRG2 vs. TRG3 in all subgroups). Specifically, we performed subgroups analysis based on treatment regimens (**Supplementary Table S1 and S2**). Based on the results of **Table 2**, the interaction analysis between regimens and TRGs demonstrated that all interaction terms had p-values greater than 0.05, indicating no evidence of significant interaction effects. This implies that the association between TRG and RFS is largely consistent across the different treatment regimens.

**Table 2:** Univariate and multivariate analyses of risk factors for recurrence.

Risk factors	Univariate analysis		Multivariate analysis	
	HR (95% CI)	*P* value	HR (95% CI)	*P* value
Age				
<60	Ref		Ref	
≥60	0.54 (0.30 ~ 0.96)	0.036	0.56 (0.31 ~ 1.02)	0.056
Gender				
female	Ref			
male	1.02 (0.44 ~ 2.37)	0.968		
BMI				
<18.5	Ref			
18.5~23.9	1.00 (0.24 ~ 4.16)	0.995		
>23.9	0.71 (0.16 ~ 3.11)	0.710		
Therapy regimen				
TACE	Ref			
TACE+TKI	1.44 (0.73 ~ 2.84)	0.294		
TACE+TKI+ICI	0.85 (0.46 ~ 1.56)	0.594		
TACE+Bev+ICI	0.65 (0.19 ~ 2.19)	0.488		
BCLC stage				
B	Ref			
C	1.13 (0.68 ~ 1.90)	0.633		
PVTT				
no	Ref			
yes	1.22 (0.70 ~ 2.14)	0.480		
HVTT				
no	Ref			
yes	1.29 (0.47 ~ 3.57)	0.625		
HBV-DNA				
-	Ref			
+	0.93 (0.56 ~ 1.57)	0.930		
Pre-AFP (ng/mL)				
<400	Ref			
≥400	0.99 (0.59 ~ 1.66)	0.969		
Pre-PIVKA-II (mAU/mL)				
<400	Ref			
≥400	1.04 (0.59 ~ 1.81)	0.899		
Pre-tumor size (cm)				
<5	Ref			
5~10	1.56 (0.77 ~ 3.19)	0.220		
>10	1.03 (0.47 ~ 2.24)	0.950		
Pre-tumor number				
single	Ref			
multiple	1.04 (0.61 ~ 1.77)	0.884		
Post-tumor size (cm)				
<5	Ref			
5~10	0.94 (0.52 ~ 1.69)	0.830		
>10	1.19 (0.61 ~ 2.32)	0.619		
Post-tumor number				
single	Ref			
multiple	1.40 (0.80 ~ 2.42)	0.236		
mRECIST				
PR	Ref			
SD	0.68 (0.17 ~ 2.84)	0.602		
Operation interval (every week)*	0.98 (0.95 ~ 1.00)	0.104		
AFP decrease to normal interval (every week)	1.00 (1.00 ~ 1.01)	0.176		
TRG				
1a	Ref		Ref	
1b	1.89 (0.51 ~ 7.03)	0.345	2.20 (0.55 ~ 8.82)	0.266
2	4.50 (1.60 ~ 12.65)	0.004	**4.25 (1.37 ~ 13.17)**	**0.012**
3	5.49 (2.15 ~ 14.02)	<0.001	**6.20 (2.11 ~ 18.26)**	**<.001**
MVI				
no	Ref		Ref	
yes	2.10 (1.23 ~ 3.58)	0.007	1.47 (0.81 ~ 2.67)	0.205
Satellite nodules				
no	Ref		Ref	
yes	2.08 (1.18 ~ 3.65)	0.011	1.43 (0.79 ~ 2.59)	0.231
Cirrhosis				
no	Ref			
yes	1.26 (0.75 ~ 2.11)	0.389		
Resection margin (cm)				
≥1	Ref			
<1	1.26 (0.73 ~ 2.17)	0.400		
IHC-AFP positive				
-	Ref			
+	1.05 (0.55 ~ 2.00)	0.885		
IHC-GPC3 positive				
-	Ref			
+	1.43 (0.64 ~ 3.18)	0.379		
IHC-GS positive				
-	Ref			
+	1.06 (0.60 ~ 1.85)	0.845		
IHC-CD34 positive				
-	Ref			
+	1.44 (0.69 ~ 2.97)	0.330		

*The time interval between the date of systemic treatment and the date of surgical resection.

BMI, body mass index; TACE, transcatheter arterial chemoembolization; TKI, tyrosine kinase inhibitors; ICI, immune checkpoint inhibitors; Bev, Bevacizumab; BCLC, Barcelona Clinic Liver Cancer; PVTT, portal vein tumor thrombus; HVTT, hepatic vein tumor thrombus; Pre-, pre-therapy; Post-, post therapy, but before surgery; AFP, Alpha fetoprotein; PIVKA-II, protein induced by vitamin K absence-II; mRECIST, modified response evaluation criteria in solid tumors; PR, partial response; SD, stable disease; MVI, microvascular invasion; IHC, immunohistochemistry; GPC3, glypican 3, GS, glutamine synthetase. The bold values are statistically significant.

### Short and long term outcomes based on TRG classification

Given the significant prognostic impact of TRG, we compared patient characteristics across TRG groups (**Table 1**). The average body mass index (BMI) was approximately 23 in most groups, except for the TRG2 group, which had a lower average BMI of 21.5. The distribution of BCLC stages varied among groups, with the TRG3 group having the highest proportion of BCLC-B patients (67.3%), while the TRG1a group included the most BCLC-C patients (62.1%, *P* = 0.041). Among BCLC-C patients, the characteristics of major vascular invasion did not differ significantly. Notably, the TRG1a group had the highest median AFP level before conversion therapy (1,210 ng/mL, *P* = 0.029), which decreased to the lowest post-treatment level (2.5 ng/mL, *P* = 0.001), indicating a marked response. Regarding HCC etiology, the pre-treatment HBV DNA positivity rate did not differ significantly among groups. However, after antiviral treatment, the positivity rates decreased significantly in all groups except the TRG3 group (35.3% to 32.7%, *P* = 0.003). The initial proportion of multiple tumors was comparable among groups. After conversion therapy, most patients exhibited tumor shrinkage, resulting in a reduction from multiple to single lesions, although this change was not significant in the TRG2 group (60.9% to 52.2%). According to mRECIST, 10 patients were evaluated as achieving radiological complete response (CR); however, pathological examination confirmed pCR (TRG1a) in 29 patients.

**Table 3** summarizes perioperative and pathological characteristics by TRG group. The operation interval (time from initiation of systemic therapy to surgical resection) was longest in the TRG1a group (median 23.7 weeks, *P* = 0.009). The time to AFP normalization did not differ significantly among groups (*P* = 0.565). Only a small proportion of patients in each group underwent laparoscopic resection, with the majority (> 80%) undergoing open surgery (*P* = 0.411). Intraoperative parameters, including operative time (*P* = 0.838) and blood loss (*P* = 0.184), were comparable among groups. The median postoperative hospital stay was approximately 6 days across all groups (*P* = 0.455). The incidence of postoperative complications, such as liver failure, bile leakage, and hemorrhage, did not differ significantly. However, notable differences were observed in pathological findings: the TRG2 group had a higher proportion of satellite nodules (47.8%, *P* = 0.013), and the TRG3 group had the highest rate of MVI (*P* = 0.001). Immunohistochemical staining revealed that the TRG1a group had the lowest positive rates for AFP, GPC3, GS, and CD34. The incidence of grade ≥ 3 adverse events during the conversion therapy period of patients are shown in **Supplementary Table S3**, and the results indicated no significant differences among all TRG groups.

**Table 3:** Perioperative and pathological characteristics of patients according to TRG classification.

Variable	Total (n=117)	TRG 1a (n=29)	TRG 1b (n=13)	TRG 2 (n=23)	TRG 3 (n=52)	*P* value
Operation interval (weeks)*	24.8 (5-237)	23.7 (8-59)	18.7 (8-48)	20.9 (8-78)	14.7 (5-237)	**0.009**
AFP decrease to normal interval (weeks)	32.7 (4-244)	22.6 (7-79)	20.7 (4-52)	24.3 (13-44)	25.6 (4-244)	0.565
Surgical procedure						0.411
LLR	13 (11.1)	5 (17.2)	0 (0)	2 (8.7)	6 (11.5)	
OLR	104 (88.9)	24 (82.8)	13 (100)	21 (91.3)	46 (88.5)	
Operation time (min)	237 (120-650)	210 (120-337)	252 (140-332)	205 (158-440)	223 (121-650)	0.838
Pringle maneuver	107 (91.5)	27 (93.1)	13 (100)	22 (95.7)	45 (86.5)	0.716
Blood loss (mL)	469 (50-9000)	300 (80-1000)	400 (100-600)	400 (100-1000)	300 (50-9000)	0.184
Transfusion	21 (17.9)	1 (3.4)	4 (30.8)	6 (26.1)	10 (19.2)	0.083
POLS (days)	6 (1-80)	6 (4-80)	6 (2-12)	6 (4-34)	6 (1-35)	0.455
Complications						
Liver failure	34 (29.1)	6 (20.7)	6 (46.2)	7 (30.4)	15 (28.8)	0.418
Hemorrhage	10 (8.5)	2 (6.9)	1 (7.7)	2 (8.7)	5 (10)	0.971
Bile leakage	2 (1.7)	0 (0)	0 (0)	1 (4.3)	1 (2.0)	0.642
Pneumonia	23 (19.7)	8 (27.6)	2 (15.4)	5 (21.7)	8 (16.0)	0.624
Incision infection	2 (1.7)	1 (3.4)	0 (0)	0 (0)	1 (2.0)	0.764
ICU	6 (5.1)	1 (3.4)	1 (7.7)	1 (4.3)	3 (5.8)	0.932
Others	3 (2.6)	2 (6.9)	0 (0)	0 (0)	1 (1.9)	0.360
Tumor differentiation						0.172
Well/Moderate	47 (58.0)	0 (0)	9 (75.0)	9 (42.9)	29 (60.4)	
Poor	34 (42.0)	0 (0)	3 (25.0)	12 (57.1)	19 (39.6)	
Resection margin (cm)	0.7 (0.1-5.0)	0.5 (0.1-2.7)	0.5 (0.1-3.6)	0.5 (0.1-3.0)	0.5 (0.1-5.0)	0.942
Satellite nodules	28 (23.9)	3 (10.3)	2 (15.4)	11 (47.8)	12 (23.1)	**0.013**
Cirrhosis	61 (52.1)	13 (44.8)	7 (53.8)	15 (65.2)	26 (50.0)	0.511
MVI	35 (29.9)	2 (6.9)	2 (15.4)	6 (26.1)	25 (48.1)	**0.001**
IHC-AFP	11 (9.4)	0 (0)	0 (0)	2 (12.5)	9 (25.0)	0.334
IHC-GPC3	68 (58.1)	1 (3.4)	7 (53.8)	21 (91.3)	39 (75.0)	**0.000**
IHC-GS	47 (40.2)	0 (0)	9 (69.2)	12 (52.2)	26 (50.0)	**0.002**
IHC-CD34	59 (50.4)	1 (3.4)	6 (46.2)	15 (65.2)	37 (71.2)	**0.000**
Mortality with 90 days	3 (2.6)	0 (0)	0 (0)	1 (4.3)	2 (4.2)	0.638

*The time interval between the date of systemic treatment and the date of surgical resection.

AFP, Alpha fetoprotein; LLR, laparoscopic liver resection; OLR, open liver resection; POLS, postoperative length of stay; ICU, intensive care unit; MVI, microvascular invasion; IHC, immunohistochemistry; GPC3, glypican 3, GS, glutamine synthetase. The bold values are statistically significant.

**Figure 2:**
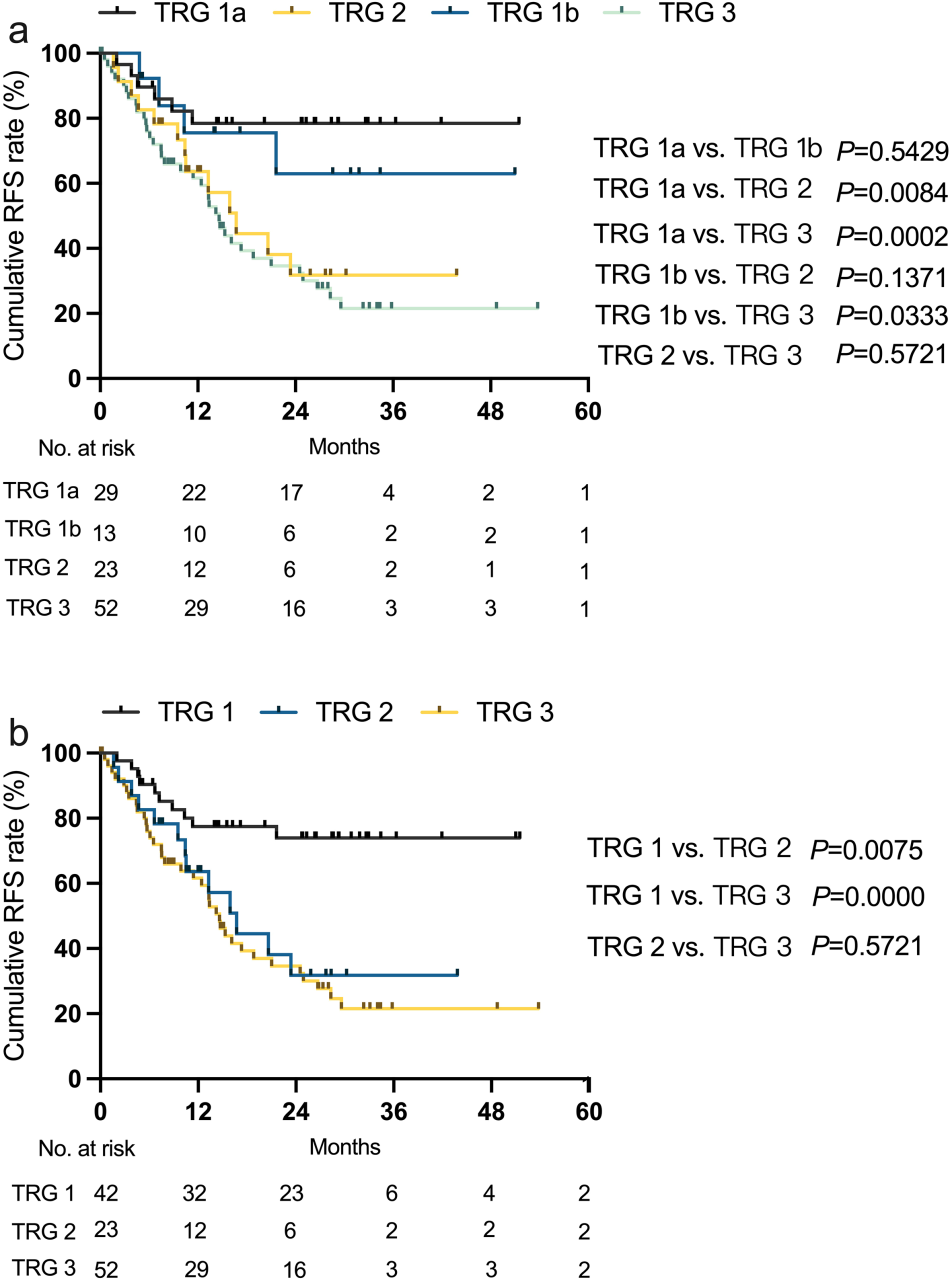
Kaplan-Meier analysis of recurrence-free survival (RFS) for patients according to tumor regression grade (TRG) classification. *TRG1 (n=42); TRG1a (n=29); TRG1b (n=13); TRG2 (n=23); TRG3 (n=52)*.

Postoperative recurrence outcomes are shown in **Table 4** and **Supplementary Figure S2**. The median RFS was not reached in the TRG1a and TRG1b groups, whereas the median RFS was 16.7 and 14.6 months in the TRG2 and TRG3 groups, respectively. During a median follow-up period of 19.3 (95% CI: 18.4 - 20.3) months, postoperative recurrence occurred in 20.7%, 30.8%, 56.5%, and 69.2% of patients in the TRG1a, TRG1b, TRG2, and TRG3 groups, respectively (*P* < 0.001). The most common site of recurrence was intrahepatic across all groups; however, the TRG3 group had the highest probability of distant recurrence (19.2%). Additionally, two cases of peritoneal metastasis were observed in the TRG2 group. Kaplan-Meier curves for RFS are shown in **Figure 2**, demonstrating significantly better RFS in the TRG1a and TRG1b groups compared to the TRG2 and TRG3 groups. A direct comparison RFS between the TRG1a and TRG1b groups revealed no statistically significant difference (Log-rank test, *P* = 0.5429). Given the lack of a significant survival disparity and the shared clinical characteristic of having minimal residual tumor burden (≤ 10%), we combined these groups into a single TRG1 category for subsequent analyses of an optimal response to conversion therapy.

**Table 4:** Recurrence patterns according to TRG classification.

Variable	TRG 1a (n=29)	TRG 1b (n=13)	TRG 2 (n=23)	TRG 3 (n=52)	*P* value
Total, n (%)	6 (20.7)	4 (30.8)	13 (56.5)	36 (69.2)	0.000
Liver only	5 (17.2)	4 (30.8)	7 (30.4)	24 (46.2)	
Liver & distant	1 (3.4)	0 (0)	0 (0)	2 (3.8)	
Distant	0 (0)	0 (0)	4 (17.4)	10 (19.2)	
Peritoneal	0 (0)	0 (0)	2 (8.7)	0 (0)	
RFS, median mo. (range)	NR	NR	16.7 (10.5-22.9)	14.6 (12.1-17.1)	0.001

NR, not reached; RFS, recurrence-free survival. The bold values are statistically significant.

### Risk factors for achieving TRG1 after conversion therapy

Given the favorable prognosis associated with TRG1(a&b), we further analyzed factors predictive of achieving this status. Multivariate logistic regression analysis (**Table 5**) identified that male gender [OR = 3.44; 95% CI (1.71 ~ 6.90), *P* < 0.001], conversion regimen comprising TACE combined with targeted therapy and immunotherapy (*P* < 0.001), pre-treatment tumor diameter of 5–10 cm [OR = 2.31; 95% CI (1.21 ~ 4.44), *P* = 0.012], and shorter time to AFP normalization [OR = 0.97; 95% CI (0.96 ~ 0.98), *P* < 0.001] were independent predictors for achieving TRG1 status.

**Table 5:** Univariate and multivariate analyses of risk factors for TRG1(a&b).

Risk factors	Univariate analysis		Multivariate analysis	
	OR (95% CI)	*P* value	OR (95% CI)	*P* value
Age: ≥60 vs.<60 years	1.39 (0.98 ~ 1.98)	0.068		
Gender: male vs. female	3.44 (1.71 ~ 6.90)	<0.001	**3.44 (1.71 ~ 6.90)**	**<0.001**
BMI				
<18.5	Ref			
18.5~23.9	1.59 (0.57 ~ 4.46)	0.380		
>23.9	2.00 (0.70 ~ 5.75)	0.198		
Therapy regimen				
TACE	Ref			
TACE+TKI	2.72 (1.50 ~ 4.94)	0.001	**3.22 (1.62 ~ 6.41)**	**<.001**
TACE+TKI+ICI	7.73 (4.73 ~ 12.64)	<.001	**6.71 (3.91 ~ 11.51)**	**<.001**
TACE+Bev+ICI	10.20 (5.04 ~ 20.63)	<.001	**15.03 (6.15~ 36.78)**	**<.001**
BCLC stage: C vs. B	2.05 (1.45 ~ 2.92)	<.001	1.15 (0.63 ~ 2.10)	0.638
PVTT: yes vs. no	2.50 (1.75 ~ 3.56)	<.001	1.56 (0.85 ~ 2.88)	0.150
HBV-DNA: + vs. -	1.48 (1.06 ~ 2.08)	0.023	1.39 (0.90 ~ 2.16)	0.142
Pre-AFP: ≥400 vs. <400 ng/mL	1.65 (1.17 ~ 2.32)	0.004	1.50 (0.98 ~ 2.32)	0.064
Pre-PIVKA-II: ≥400 vs. <400 mAU/mL	1.45 (0.99 ~ 2.13)	0.058		
Pre-tumor size (cm)				
<5	Ref		1.00 (Reference)	
5~10	1.96 (1.17 ~ 3.27)	0.010	**2.31 (1.21 ~ 4.44)**	**0.012**
>10	2.45 (1.45 ~ 4.12)	<.001	1.95 (0.97 ~ 3.94)	0.062
Pre-tumor number: multiple vs. single	1.14 (0.81 ~ 1.62)	0.444		
Operation interval (every week)*	1.00 (0.99 ~ 1.01)	0.960		
AFP decrease to normal interval (every week)	0.98 (0.98 ~ 0.99)	<.001	0.97 (0.96 ~ 0.98)	**<.001**

*The time interval between the start of systematic treatment and the time of received surgery.

BMI, body mass index; TACE, transcatheter arterial chemoembolization; TKI, tyrosine kinase inhibitors; ICI, immune checkpoint inhibitors; Bev, Bevacizumab; BCLC, Barcelona Clinic Liver Cancer; PVTT, portal vein tumor thrombus; HVTT, hepatic vein tumor thrombus; Pre-, pre-therapy; AFP, Alpha fetoprotein; PIVKA-II, protein induced by vitamin K absence-II. The bold values are statistically significant.

## Discussion

This study demonstrates that the pathological TRG status following successful conversion therapy has a significant prognostic impact on HCC and advanced TRG grades predict poorer RFS. Furthermore, male gender, tumors measuring 5–10 cm, the use of TACE combined with targeted therapy and immunotherapy, and rapid normalization of AFP levels were identified as significant predictors for achieving optimal TRG1(a&b) status. Our finding that TRG1a (pCR) and TRG1b (≤ 10% residual tumor) present a similar and favorable prognosis challenges a strict dichotomy but aligns with the concept of MPR as a critical therapeutic endpoint in oncology. The biological rationale for combining these groups is multifactorial. Firstly, the threshold of ≤ 10% viable tumor may represent a critical biological tipping point. Below this level, the host’s immune system, potentially activated by the immunotherapy, may be capable of controlling or eradicating the minimal residual disease, thereby abrogating the clinical difference between 0% and 10% residual tumor. Secondly, from a clinical perspective, both states (0% and ≤10% residual tumor) represent an profound response to therapy, vastly superior to the outcomes seen with higher residual tumor burdens (TRG2/3). This suggests that the goal of conversion therapy could be expanded from pursuing only pCR to achieving MPR (defined here as TRG1), a target that may be more frequently attainable and equally important on long-term survival. Therefore, combining TRG1a and TRG1b into an optimal response group provides a clinically meaningful stratification that effectively identifies patients with the best postoperative prognosis.

Recent advances in targeted therapy and immunotherapy have enabled some patients with advanced HCC to qualify for radical resection. Pathological assessment of resected specimens after conversion therapy reveals varying degrees of tumor necrosis, reflecting treatment efficacy. Our study, based on real-world data, indicates that achieving less than 10% residual tumor (TRG1) significantly improves RFS, aligning with the primary goal of conversion therapy (24). The proportion of viable tumor in the resected specimen directly reflects the effectiveness of conversion treatment. However, assessing tumor response based solely on imaging (mRECIST) or tumor markers can be challenging (25, 26), as evidenced by the discrepancy between radiological CR and pathological CR in our cohort (**Supplementary Table S4**). While mRECIST criteria, based on the absence of enhancing tumor tissue on imaging, are important for non-invasive treatment monitoring, they primarily reflect macroscopic vascularity and tumor architecture. In contrast, pathological examination reveals the microscopic reality of tumor viability. This discrepancy indicates the inherent limitations of cross-sectional imaging in detecting microscopic residual disease or scattered viable tumor cells in treatment-induced fibrosis and necrosis. Conversely, some tumors judged as partial response or stable disease by mRECIST were found to have no viable tumor (pCR) upon resection, possibly due to non-enhancing yet necrotic tumor masses that persist on imaging. This finding reinforces the concept that pathological assessment remains the gold standard for evaluating true tumor response following conversion therapy and the need for more accurate response assessment tools, such as novel imaging techniques like FAPI PET-CT (27), though their widespread use remains limited by cost and availability.

Interestingly, within our cohort, the TRG1(a&b) group contained a higher proportion of BCLC-C patients yet exhibited superior outcomes. Although the sample size limits the strength of this observation, it suggests that in the era of conversion therapy, initial tumor stage should not be the sole determinant of long-term survival potential. Successful downstaging followed by conversion surgery can lead to excellent outcomes, as previously reported (28).

On the other hand, our findings underscore the importance of striving for TRG1 status, irrespective of initial stage, and identify several predictive factors. The association between male gender and higher likelihood of TRG1 suggests potential gender-specific differences in treatment response, warranting further investigation. The correlation between moderately sized tumors (5–10 cm) and better pathological response may be attributed to more effective drug delivery and targeting compared to very large or diffuse lesions. While a smaller tumor might form the limited blood supply system thereby reducing the efficiency of drug administration, larger lesions often accompany with intra-tumor necrosis or hemorrhage and thereby increasing the difficulty of drug targeting process. Furthermore, the results also highlighted that the combine TACE with targeted therapies and immunotherapy was independently associated with achieving TRG1 status. Theoretically, multimodal treatments can enhance the therapeutic effect by simultaneously attacking the tumor through different mechanisms (29, 30). Similar evidence has been reported by proving the survival benefit of combining TACE with other systemic therapies for advanced HCC patients, suggesting that integrating multiple therapeutic modalities could be a key strategy in improving conversion therapy outcomes (16, 31).

Besides, rapid normalization of AFP levels emerged as another strong predictor. In general, a quicker normalization of AFP level reflects the sensitivity of certain treatments to HCC lesions. Our finding that rapid AFP normalization is a strong independent predictor for achieving TRG1 status aligns with a growing body of literature affirming the role of AFP as a dynamic, quantitative biomarker for monitoring therapeutic efficacy in the context of atezolizumab-bevacizumab therapy (26, 32) and other ICI-based treatments (33). The correlation between serological (AFP) and histological (TRG) response further underscores the biological validity of both metrics and supports the use of on-treatment AFP levels to guide the timing of surgical intervention in conversion therapy. However, as highlighted in a recent comprehensive review by Yu et al., the future of HCC management lies in moving beyond single biomarkers towards integrated, multi-parametric approaches (34). Our findings, which focus on AFP and PIVKA-II, align with this vision by demonstrating that a serological marker (AFP) can serve as a surrogate for a sophisticated pathological endpoint (TRG). Looking forward, the biomarker landscape is rapidly expanding to include novel circulating factors (e.g., glypican 3, PD-L1 expression, tumor-infiltrating lymphocytes, circulating tumor cells, circulating tumor DNA), genomic and transcriptomic signatures from liquid biopsies, and radiologic features from medical imaging. These novel biomarkers hold the promise of capturing the complex tumor heterogeneity and immune microenvironment, which are critical determinants of response to targeted and immunotherapeutic agents used in conversion therapy. Therefore, while AFP remains a cornerstone in current clinical practice, our work sets the stage for future research that combines traditional markers like AFP and PIVKA-II with these emerging biomarkers. Such integrated models could potentially predict pathological responses like TRG non-invasively and with greater accuracy, ultimately enabling more personalized and effective treatment strategies for patients with intermediate-advanced HCC.

Pathological findings further supported the prognostic value of TRG. The higher incidence of adverse features like satellite nodules in the TRG2 group and MVI in the TRG3 group likely contributed to their higher recurrence rates (35, 36). The lower positive rates of AFP, GPC3, GS, and CD34 in the TRG1a group further indicate a lower residual tumor burden and a more favorable tumor biology responded to conversion therapy. On the other hand, the consistency of short-term postoperative recovery outcomes, such as surgical time, intraoperative bleeding, and hospital stay across the TRG groups suggest that the improved RFS in the TRG1 groups was attributable to the biological effectiveness of conversion therapy rather than perioperative factors.

This study has several limitations inherent to its retrospective, single-center design, including potential selection bias and limited generalizability. The decision to initiate conversion therapy was made by a MDT for patients who were deemed to have sufficient liver reserve and performance status to potentially tolerate aggressive multimodal treatment. And more critically, only those patients who demonstrated sufficient tumor control or downstaging to render the lesion technically and oncologically resectable proceeded to surgery. This creates a selected cohort of ‘conversion successes’. Consequently, our findings on the prognostic impact of TRG are most directly applicable to this specific population of patients who have successfully navigated the conversion therapy pathway and undergone resection. While this selection process limits the broad applicability of our results, it precisely defines the clinical context in which pathological TRG assessment provides the most actionable prognostic information. Although our multivariate analysis identified ‘TACE combined with targeted therapy and immunotherapy’ as an independent predictor for achieving TRG1, the heterogeneity of specific agents within these broad categories must be acknowledged as a potential confounding factor. The sample size precluded a definitive analysis of each drug combination’s impact on TRG. Therefore, our findings regarding predictors for TRG1 should be interpreted with caution and require validation in larger, more homogeneously treated cohorts. Additionally, the pathological assessment has long been developed in colorectal liver metastasis (37), lung cancer (38), gastric carcinoma (17), and breast cancer (39), showing that histological response to preoperative systemic therapy is significantly correlated with patients’ long-term survival. It is important to note that the TRG assessment in this study was based on the Becker system, which was originally developed for gastric cancer (17). While this system provides a standardized and practical framework for quantifying tumor regression, the specific cut-off values and histological features most prognostic for HCC after conversion therapy may differ and warrant further validation. The unique pathological characteristics of HCC, such as its propensity for vascular invasion and specific stromal responses, might not be fully captured by this system. Future studies should aim to develop and validate a TRG system specifically tailored for HCC in the era of modern systemic therapy. Despite these, our study provides a valuable and readily applicable pathological framework for predicting recurrence and guiding management in HCC patients undergoing conversion therapy.

In conclusion, our study demonstrates that achieving TRG1(a&b) status (0 - 10% residual tumor) following conversion therapy is associated with significantly improved RFS in patients with intermediate-advanced HCC. Male gender, tumors measuring 5–10 cm, the combination of TACE with targeted and immunotherapy as conversion regimen, and rapid normalization of AFP levels are key predictors for achieving this favorable pathological response. This provides significant evidence on identifying the most favorable populations for conversion therapy and developing more tailored postoperative management strategies to improve oncological outcomes. To translate our findings into clinical practice, future validation in large-scale, prospective, multicenter studies is essential. Such studies should aim to standardize the pathological assessment of TRG for HCC, potentially refining the cut-off values for optimal prognostication. Furthermore, the development of integrated prognostic models that combine TRG with other critical variables, such as preoperative imaging features and serological biomarkers, is of significance to provide a more comprehensive prediction of individual patient outcomes. Overall, while our study establishes the prognostic value of TRG, it also paves the way for a new research paradigm focused on validating and predicting this key pathological endpoint through collaborative, multi-institutional efforts and the integration of novel technologies.

## Data Availability

The raw data supporting the conclusions of this article will be made available by the authors, without undue reservation.
