# Supplementary material for: Tumor regression grade predicts recurrence-free survival in intermediate-advanced hepatocellular carcinoma after conversion therapy
Source: Front Immunol. 2025 Oct 30;16:1704239. doi: 10.3389/fimmu.2025.1704239 (PMC12611931; doi:10.3389/fimmu.2025.1704239)
Supplement: Supplementary file 1 [file Table1.docx]

| **Table S1. Baseline characteristics stratified by primary conversion therapy regimen** | | | | | | |
| --- | --- | --- | --- | --- | --- | --- |
| Variable | Total (n=117) | TACE  (n=39) | TACE+TKI  (n=21) | TACE+TKI+ICI  (n=47) | TACE+Bev+ICI  (n=10) | *P* value |
| Age, (years) | 53 (20-85) | 54 (30-77) | 56 (20-85) | 55 (28-77) | 51 (34-67) | 0.642 |
| Male | 104 (88.9) | 34 (87.2) | 18 (85.7) | 45 (95.7) | 7 (70.0) | 0.103 |
| BMI | 23.1 (17.0-36.4) | 22.9 (18.8-36.4) | 23.1 (18.7-27.1) | 23.1 (17.0-29.1) | 21.9 (18.4-27.0) | 0.397 |
| Medical history |  |  |  |  |  |  |
| HBP | 25 (21.4) | 12 (30.8) | 2 (9.5) | 9 (19.1) | 2 (20.0) | 0.855 |
| COPD | 6 (5.1) | 2 (5.1) | 1 (4.8) | 3 (6.4) | 0 (0) | 0.874 |
| DM | 16 (13.7) | 6 (15.4) | 2 (9.5) | 6 (12.8) | 2 (20.0) | 0.855 |
| Portal hypertension | 38 (32.5) | 9 (23.1) | 8 (38.1) | 18 (38.3) | 3 (30.0) | 0.453 |
| Hypersplenism | 26 (22.2) | 4 (10.3) | 6 (28.6) | 13 (27.7) | 3 (30.0) | 0.181 |
| BCLC stage |  |  |  |  |  | 0.004 |
| B | 62 (53.0) | 30 (76.9) | 9 (42.9) | 19 (40.4) | 4 (40.0) |  |
| C | 55 (47.0) | 9 (23.1) | 12 (57.1) | 28 (59.6) | 6 (60.0) |  |
| PVTT classification |  |  |  |  |  | 0.932 |
| Vp1 | 3 (2.6) | 0 (0) | 1 (8.3) | 1 (4.2) | 1 (16.7) |  |
| Vp2 | 7 (5.9) | 0 (0) | 3 (25.0) | 3 (12.5) | 1 (16.7) |  |
| Vp3 | 20 (17.1) | 3 (60.0) | 5 (41.7) | 10 (41.7) | 2 (33.3) |  |
| Vp4 | 10 (8.5) | 1 (20.0) | 1 (8.3) | 7 (29.2) | 1 (16.7) |  |
| HVTT | 7 (5.9) | 1 (20.0) | 2 (16.7) | 3 (12.5) | 1 (16.7) |  |
| Pre-AFP (ng/mL) | 473.6 (1-551261) | 268 (1-33973) | 125 (2.6-174903) | 584 (2-77992) | 7637 (129-551261) | 0.001 |
| Pre-PIVKA-II (mAU/mL) | 3216.0 (0-75000) | 2106 (0-75000) | 2183 (63-27025) | 6231 (19-75000) | 4413 (14-75000) | 0.586 |
| Pre-HBVDNA+ | 50 (42.7) | 15 (38.5) | 10 (47.6) | 18 (38.3) | 7 (70.0) | 0.248 |
| Pre-tumor size (cm) | 8.7 (1-20) | 7.6 (1-20) | 7.0 (1.3-12.9) | 9.5 (1.4-19.4) | 9.9 (3.8-13.0) | 0.303 |
| Pre-tumor number |  |  |  |  |  | 0.741 |
| single | 47 (40.2) | 16 (41.0) | 7 (33.3) | 21 (44.7) | 3 (30.0) |  |
| multiple | 70 (59.8) | 23 (59.0) | 14 (66.7) | 26 (55.3) | 7 (70.0) |  |
| Post-AFP (ng/mL) | 4.2 (0-2286) | 6.3 (1.1-1210) | 4.3 (0.9-1071) | 3.4 (0-2286) | 6.2 (0.9-62.2) | 0.265 |
| Post-PIVKA-II (mAU/mL) | 27.0 (0-645) | 26.5 (0-645) | 38 (11-368) | 25 (11-301) | 31 (16-461) | 0.265 |
| Post-HBVDNA+ | 21 (17.9) | 12 (30.8) | 4 (719.0) | 5 (10.6) | 0 (0) | 0.041 |
| Post-tumor size (cm) | 6.9 (0.6-23) | 8.0 (0.8-23) | 5.0 (0.6-12.0) | 6.0 (0.6-13.5) | 6.9 (2.0-12.2) | 0.091 |
| Post-tumor number |  |  |  |  |  | 0.922 |
| single | 85 (72.6) | 29 (74.4) | 15 (71.4) | 33 (70.2) | 8 (80.0) |  |
| multiple | 32 (27.4) | 10 25.6) | 6 (28.6) | 14 (29.8) | 2 (20.0) |  |
| mRECIST |  |  |  |  |  | 0.038 |
| CR | 10 (8.5) | 1 (2.6) | 3 (14.3) | 6 (12.8) | 0 (0) |  |
| PR | 38 (32.5) | 9 (23.1) | 4 (19.0) | 19 (40.4) | 6 (60.0) |  |
| SD | 69 (59.0) | 29 (74.4) | 14 (66.7) | 22 (46.8) | 4 (40.0) |  |
| BMI, body mass index; HBP, high blood pressure; COPD, chronic obstructive pulmonary disease; DM, diabetes mellitus; BCLC, Barcelona Clinic Liver Cancer; PVTT, portal vein tumor thrombus; HVTT, hepatic vein tumor thrombus; Pre-, pre-therapy; Post-, post-therapy, but before surgery; AFP, Alpha fetoprotein; PIVKA-II, protein induced by vitamin K absence-II; TACE, transcatheter arterial chemoembolization; TKI, tyrosine kinase inhibitors; ICI, immune checkpoint inhibitors; Bev, Bevacizumab; mRECIST, modified response evaluation criteria in solid tumors; CR, complete response; PR, partial response; SD, stable disease. | | | | | | |

| **Table S2. Interaction analysis between regimens and TRGs based on the model in Table 2** | | | | |
| --- | --- | --- | --- | --- |
| Risk factors | Univariate analysis | | Multivariate analysis | |
|  | HR (95% CI) | *P* value | HR (95% CI) | *P* value |
| Interactions analysis |  |  |  |  |
| TACE * TRGs/Regimens * TRG1a | Ref |  | Ref |  |
| TACE+TKI * TRG1b | 0.00 (0.00 ~ Inf) | 0.996 | 0.00 (0.00 ~ Inf) | 0.997 |
| TACE+TKI+ICI * TRG1b/TACE+TKI * TRG2 | 1.43 (0.64 ~ 3.18) | 0.382 | 3.29 (0.63 ~ 17.36) | 0.160 |
| TACE+Bev+ICI * TRG1b/TACE+TKI * TRG3 | 3.61 (1.61 ~ 8.09) | 0.002 | 1.39 (0.54 ~ 3.60) | 0.493 |
| TACE+TKI+ICI * TRG2 | 2.02 (0.82 ~ 4.94) | 0.124 | 2.78 (0.56 ~ 13.77) | 0.211 |
| TACE+Bev+ICI * TRG2/TACE+TKI+ICI * TRG2 | 2.45 (1.13 ~ 5.31) | 0.023 | 1.18 (0.48 ~ 2.92) | 0.723 |
| TACE+Bev+ICI * TRG3 | 4.47 (1.03 ~ 19.31) | 0.045 | 2.40 (0.52 ~ 11.03) | 0.262 |
| TACE, transcatheter arterial chemoembolization; TKI, tyrosine kinase inhibitors; ICI, immune checkpoint inhibitors; Bev, Bevacizumab; TRG, tumor regression grade. | | | | |

| **Table 3. Incidence of grade ≥ 3 adverse events of patients according to TRG classification** | | | | | | |
| --- | --- | --- | --- | --- | --- | --- |
| Variable | Total (n=117) | TRG 1a (n=29) | TRG 1b (n=13) | TRG 2 (n=23) | TRG 3 (n=52) | *P* value |
| High blood pressure | 2 (1.7) | 1 (3.4) | 0 (0) | 1 (4.3) | 0 (0) | 0.457 |
| Hepatotoxicity | 4 (3.4) | 0 (0) | 0 (0) | 2 (8.7) | 2 (3.8) | 0.327 |
| Thrombocytopenia | 4 (3.4) | 0 (0) | 0 (0) | 2 (8.7) | 2 (3.8) | 0.327 |
| Myelosuppression | 1 (0.9) | 0 (0) | 0 (0) | 0 (0) | 1 (1.9) | 0.738 |
| Gingival reactions | 2 (1.7) | 0 (0) | 0 (0) | 0 (0) | 2 (3.8) | 0.467 |
| Cutaneous adverse reactions | 5 (4.3) | 2 (6.9) | 0 (0) | 0 (0) | 3 (5.8) | 0.498 |
| Bleeding events | 1 (0.9) | 1 (3.4) | 0 (0) | 0 (0) | 0 (0) | 0.382 |
| Myocarditis | 1 (0.9) | 0 (0) | 1 (7.7) | 0 (0) | 0 (0) | 0.045 |
| Hypothyroidism | 3 (2.6) | 1 (3.4) | 1 (7.7) | 0 (0) | 1 (1.9) | 0.542 |
| TRG, tumor regression grade. | | | | | | |

| **Table S4. Correlation between radiological and pathological assessment** | | | |
| --- | --- | --- | --- |
| Pathological assessment | Radiological assessment | | |
|  | CR | PR | SD |
| TRG 1a | 10 | 12 | 7 |
| TRG 1b | 0 | 5 | 8 |
| TRG 2 | 0 | 8 | 15 |
| TRG 3 | 0 | 13 | 39 |
| Pearson’s R: 0.498, P < 0.05;  Spearman Correlation: 0.438, P < 0.05.  TRG, tumor regression grade; CR, complete response; PR, partial response; SD, stable disease. | | | |


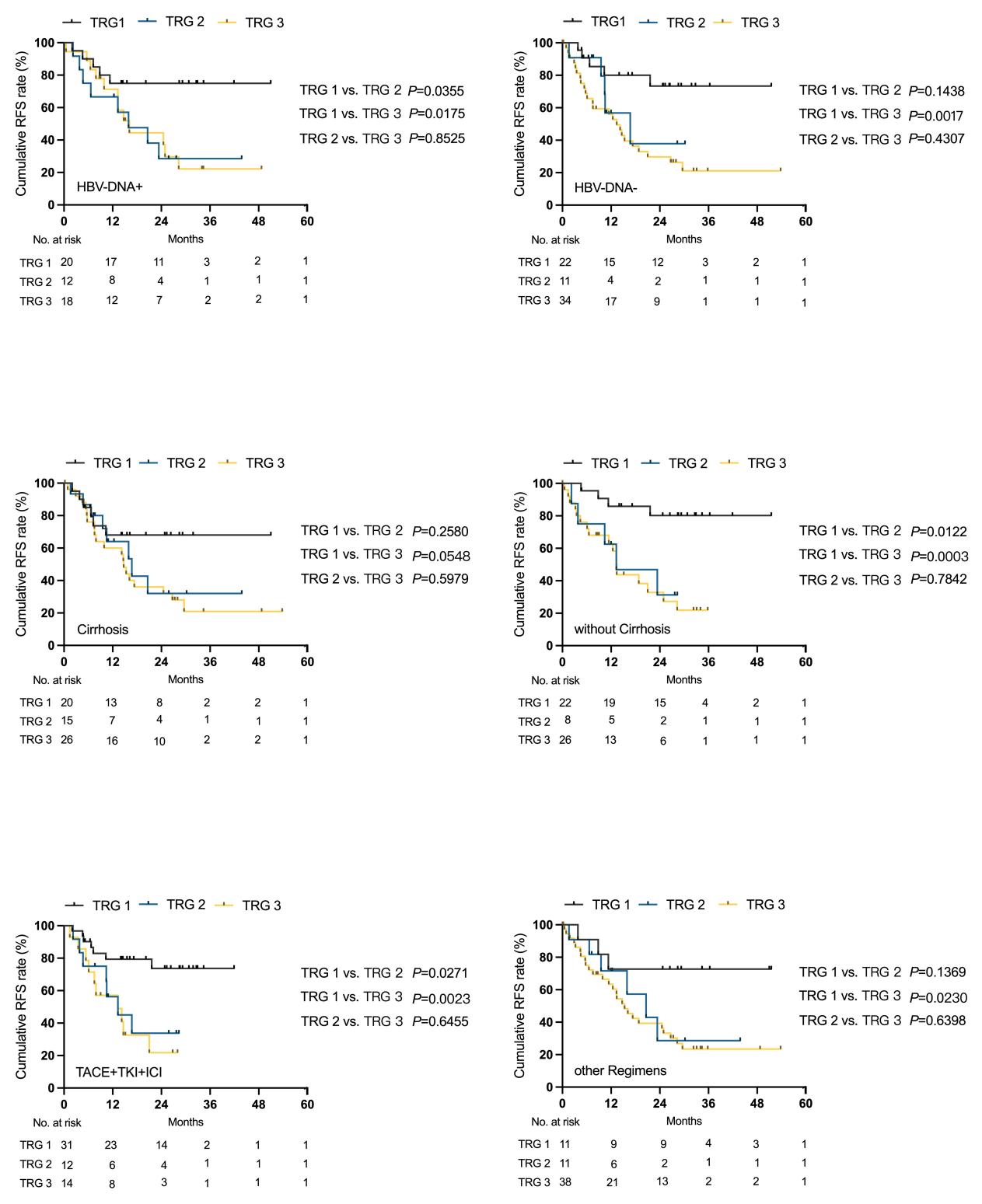


**Figure S1: Kaplan-Meier analysis of recurrence-free survival for patients according to different subgroups.** a) Kaplan–Meier analysis of RFS in patients positive for HBV DNA according to TRG classification; b) Kaplan–Meier analysis of RFS in patients negative for HBV DNA according to TRG classification; c) Kaplan–Meier analysis of RFS in patients with cirrhosis according to TRG classification; d) Kaplan–Meier analysis of RFS in patients without cirrhosis according to TRG classification; e) Kaplan–Meier analysis of RFS in patients treated with TACE+TKI+ICI according to TRG classification; f) Kaplan–Meier analysis of RFS in patients treated with other regimens according to TRG classification. *RFS, recurrence-free survival; TRG, tumor regression grade; HBV, hepatitis B virus; TACE, transcatheter arterial chemoembolization; TKI, tyrosine kinase inhibitors; ICI, immune checkpoint inhibitors.*

*
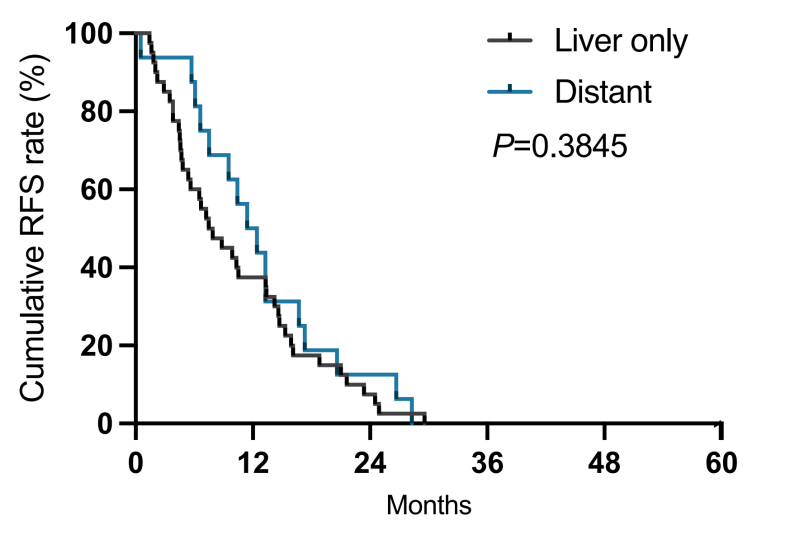
*

**Figure S2: Kaplan–Meier analysis of recurrence-free survival for patients according to different recurrent patterns.**
